# A dose response model for *Staphylococcus aureus*

**DOI:** 10.1038/s41598-021-91822-y

**Published:** 2021-06-15

**Authors:** Srikiran Chandrasekaran, Sunny C. Jiang

**Affiliations:** Civil and Environmental Engineering, University of California, Irvine, Irvine, 92697 USA

**Keywords:** Infectious diseases, Computational biology and bioinformatics, Computational models

## Abstract

Dose-response models (DRMs) are used to predict the probability of microbial infection when a person is exposed to a given number of pathogens. In this study, we propose a new DRM for *Staphylococcus aureus* (SA), which causes skin and soft-tissue infections. The current approach to SA dose-response is only partially mechanistic and assumes that individual bacteria do not interact with each other. Our proposed two-compartment (2C) model assumes that bacteria that have not adjusted to the host environment decay. After adjusting to the host, they exhibit logistic/cooperative growth, eventually causing disease. The transition between the adjusted and un-adjusted states is a stochastic process, which the 2C DRM explicitly models to predict response probabilities. By fitting the 2C model to SA pathogenesis data, we show that cooperation between individual SA bacteria is sufficient (and, within the scope of the 2C model, necessary) to characterize the dose-response. This is a departure from the classical single-hit theory of dose-response, where complete independence is assumed between individual pathogens. From a quantitative microbial risk assessment standpoint, the mechanistic basis of the 2C DRM enables transparent modeling of dose-response of antibiotic-resistant SA that has not been possible before. It also enables the modeling of scenarios having multiple/non-instantaneous exposures, with minimal assumptions.

## Introduction

The opportunistic pathogen, *Staphylococcus aureus* (henceforth SA), is carried by 10 to 20 percent of the human population^1^. It causes infections of the skin and soft tissue, but can also invade the bloodstream. Methicillin-resistant SA or MRSA, is used to describe the SA strains that are resistant to penicillinase-stable beta-lactam antibiotics such as methicillin^2^ (unless specified otherwise, SA refers to methicillin-sensitive SA). The Centers for Disease Control and Prevention (CDC) estimated 80,461 invasive infections and 11,285 deaths due to MRSA in the US in 2011^3^.

Modeling infectious disease dynamics has helped inform policy for disease management^4^. In the case of SA, past studies have investigated person-to-person transmission dynamics in hospitals^5–7^. The transmission of SA from the environment to humans has received comparatively less attention although SA has been detected in various environmental compartments^8,9^. Infectious disease models that account for environmental pathogen loads build upon the SIR (susceptible, infected, recovered) model^10–12^. In such models, the human population is split into three compartments: susceptible(S), infected(I) and recovered(R). The total population size is held constant and people move between compartments at rates defined in the model (see^11,12^ for details). Pertaining to the environment, susceptible individuals become infected individuals at a rate dependent on the bacterial load in the environment, which is tracked in a fourth compartment^11,12^. The relationship between bacterial load (dose) and the rate of a susceptible person becoming infected (showing response) is understood using dose-response models (DRMs), which are well studied in the context of quantitative microbial risk assessment (QMRA)^13^. Classical DRMs relate the dose of pathogen a person is exposed to and the probability of that person showing observable symptoms/response.

The current DRM for SA is reported by Rose and Haas^14^. It is based on the experiments of Singh and colleagues^15^, where volunteers’ forearms were inoculated with SA. Their skin was cleaned with alcohol beforehand to reduce the resident microflora and potentially enhance SA growth. Measurements of SA density on the skin (growth data) and the probability of developing lesions (dose-response data) were reported for multiple initial doses of SA. The Rose-Haas (RH) DRM begins with a quasi-mechanistic model of SA growth on skin, fitted to the growth data of Singh et al.’s experiments. This model of growth is used to compute a revised dose, which is used with the classical exponential DRM^13^ to predict the probability of a person developing lesions. The revised dose was set to the area under the SA density vs. time curve (limited to 6 days), to account for the effect of the duration of SA on the skin. We refer to this as the RH model in the rest of this study. Additional experiments were performed by Rose and Haas^14^ to elucidate what happens in the initial stages when the skin resident microflora is not removed. These conditions are assumed to better reflect a real exposure to SA, and the RH model parameters were tweaked to fit this data.

In this study, we present an alternate model of infection kinetics called the two-compartment or 2C model. Simulating this model as a stochastic process gives rise to dose-response probabilities, and we fit this model to the SA data from Singh et al. In comparison with the RH model, the 2C model has a fully mechanistic basis in the kinetics of SA growth. This allows it to be applied in cases with (1) non-instantaneous exposures^16^, (2) environment-host transmission dynamics with multiple exposures, without assuming independence between exposures and (3) antibiotic treatment and presence of an antibiotic resistant strain^17^. Simulating non-instantaneous or multiple exposures with classical DRMs may require assuming that each of the two doses are independent of each other, without accounting for the duration between the two doses. Antibiotic concentrations and an antibiotic-resistant subpopulation may affect disease outcomes (e.g, if the patient can be treated with a certain antibiotic or not)^17^. A fully mechanistic model fitted to sensitive strains can be applied to resistant strains with transparent assumptions e.g., any difference in fitness between strains can be attributed to the change in one or more parameters.

The 2C model is also able to estimate the probability of becoming a carrier—individuals who harbor the pathogen but do not show symptoms. These individuals form a significant proportion of the population in the case of SA exposure ^18^. In comparison with the RH model, and approaches in classical dose-response^19,20^, there isn’t a need for additional data on carrier response (also called infection response, in contrast with symptomatic or illness response)—the estimates are generated directly from data on symptomatic response and stochastic simulations of the 2C model.

The manuscript is organized as follows. We first introduce the 2C model, going over the associated assumptions and mathematical behaviors of the deterministic model. Then we investigate the stochastic analogue of the 2C model, which provides a method to computationally evaluate (symptomatic) dose-response and carrier probabilities. We then describe the relevant experiments and data on SA infection reported in the Singh et al. study and the Rose and Haas study^14,15^. We proceed to fit the 2C model to the experimental data and determine the unknown parameters. Finally, we close with a simple example on how this modeling approach may be applied to determine infection probabilities of MRSA in the environment.

## Results

### Deterministic model of kinetics

**Figure 1:**
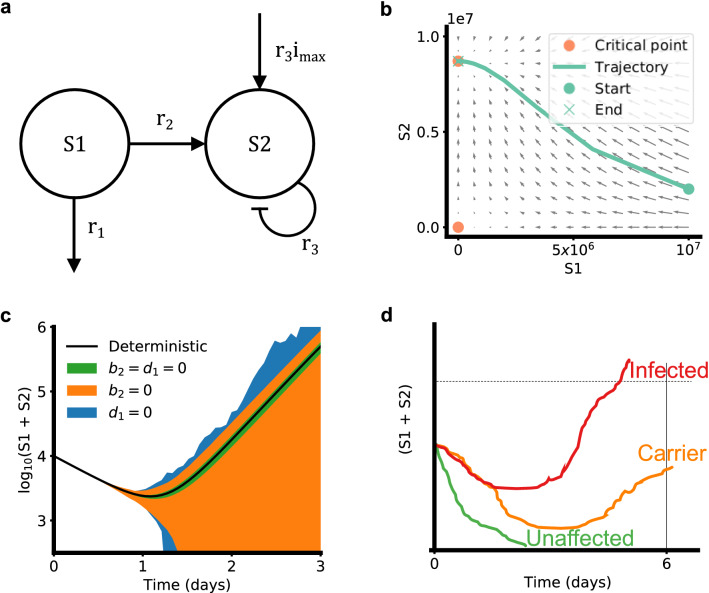
Model overview. (**a**) 2C model schematic. (**b**) Phase plot of 2C model. (**c**) Variations in 2C stochastic simulation (each region bounds mean $\pm {}$ SD of 50 simulations). (**d**) Human health outcome classification according to SA dynamics.

It has been observed that bacteria transferred to a host sometimes undergo an initial decay before growing in numbers to cause infection^15,21,22^. We hypothesize that this is due to the existence of two distinct states of the bacterium, S1 and S2 (Fig. 1a). Bacteria in S1 experience decay upon encountering the human host. This is because they are in the lag phase, adjusting to the nutrient availability in the host medium^23^. Meanwhile, the host’s immune response coupled with the lack of active cell division in the lag phase results in a net reduction of numbers. Bacteria in S1 can transition to the state S2, in which they are well adjusted to the host. The transition from S1 to S2 captures the activation of virulence genes and the range of mechanisms adopted for pathogenesis by the bacteria e.g. adhesion, invasion, etc. In this state, they exhibit density-dependent or logistic growth, which consists of the log and stationary phases. Suppose *h*(*t*) and *i*(*t*) represent the density of bacteria in S1 and S2 respectively. The differential equation representing S1-S2 dynamics is given by:

1 $$\begin{aligned} \frac{dh(t)}{dt}&= -r_1 h(t)-r_2 h(t) \end{aligned}$$

2 $$\begin{aligned} \frac{di(t)}{dt}&= r_2 h(t) + r_3 i(t) (i_{\text {max}}- i(t)) \end{aligned}$$

Here, $r_1$ (units: day$^{-1}$) is the rate of death of cells in S1. $r_2$ (units: day$^{-1}$) is the S1 to S2 transition rate. $r_3$ (units: cm$^2$/(CFU day)) is the logistic growth rate of cells in S2 and $i_{\text {max}}$ (units: CFU/cm$^2$) is the logistic carrying capacity. The $r_1 h(t)$ term captures decay in the S1 state and $r_2 h(t)$ captures transfer from S1 to S2 state. The $r_3i(t)(i_{\text {max}}{} - i(t))$ represents the density-dependent growth, since growth is slowed down at extreme values of *i*(*t*) (e.g. $i(t) = 0$ or $i(t) = i_{\text {max}}{}$) and is higher at intermediate values of *i*(*t*). $r_3$ controls the overall growth rate at all values of *i*(*t*). In contrast with the commonly used Monod model, the logistic model does not explicitly account for substrate concentration. In addition to this, other effects e.g. space limitations are implicitly captured in the density-dependent term. We call this model the two-compartment or 2C model (Fig 1a). We note that the units of $r_3$ and $i_{\text {max}}$ are in terms of two-dimensional skin surface area on which SA is being studied here.

The model has two critical points at (0, 0) and $(0, i_{\text {max}}{})$ (Fig 1b). The former is unstable whereas the latter is asymptotically stable. In other words, if the system is nudged away from the unstable critical point at (0, 0) (by addition of bacteria to the system), it will move towards the other critical point at $(0, i_{\text {max}}{})$. This system represents the density of bacteria and not their numbers, as differential equations are not restricted to integer values.

### Stochastic model of dose-response

The stochastic version of the 2C model is captured with continuous-time Markov chains (CTMC)^24^, which restrict bacterial numbers to integer values. The first order reactions associated with $r_1$ and $r_2$ in Eq. (1) are easily represented in this framework by a simple death processes^24^ with death rates $r_1$ and $r_2$ respectively. The $r_2$ term in Eq. (2) is represented by a simple birth process with birth rate $r_2$.

Interestingly, the stochastic version of the logistic growth in Eq. (2) ($r_3 i(t) (i_{\text {max}}- i(t))$) is more involved, and can be modeled using the logistic growth process^24^. In essence, the logistic growth process captures stochasticity that arises in logistic growth, when the number of species in the system is small. It consists of two birth processes (rate constants $b_1$, $b_2$) and two death processes (rate constants $d_1$, $d_2$), given by the elementary reactions defined in Table 1. The relationship between the rate constants of these elementary reactions and the rate constants of deterministic logistic growth ($r_3$ and $i_{\text {max}}$) is discussed elsewhere^24^ and shown in Eq. (3).

3 $$\begin{aligned} b_1 - d_1&= r_3i_{\text {max}}\nonumber \\ b_2 - d_2&= 2(-r_3)/A \end{aligned}$$

Here, we note that the factor 2/*A* stems from the size of the system (2D system with surface area = *A*), which needs to be accounted for in systems with second order kinetics^25^. The first order components (associated with $d_1$ and $b_1$) are density independent and reflect the death and division (birth) of cells independent of other cells. The second order components (associated with $d_2$ and $b_2$) are density-dependent and reflect the growth and death of cells as influenced by the number of cells in their neighborhood. $d_2$ can be viewed as the effect of resources competition among cells, creating an upper limit on the population size. We interpret $b_2$ as the effect of cooperation between individual SA cells, where signals from one (or more) cell(s) enhance the fitness (growth rate) of other cells e.g. by quorum sensing. This effect increases with increasing cell numbers.

These first and second order rate constants ($d_1$, $b_1$, $d_2$ and $b_2$) affect the total S1 + S2 population dynamics in interesting ways. To explore this, we start with the case where the numerical values of the deterministic rate constants ($r_1$, $r_2$, $r_3$ and $i_{\text {max}}$) are known. These numerical values along with the constraints imposed by Eq. (3) mean that among {$d_1$, $b_1$, $d_2$, $b_2$}, only two need to be picked to fully determine the behavior of the system. Picking $d_1$ and $b_2$, we examine 3 cases i) $b_2=d_1=0$ ii) $b_2 = 0$, $d_1 > 0$ and iii) $d_1 = 0$, $b_2 > 0$. For each of these cases, we examine the variance in total bacterial load (in states S1 + S2, given by $(h(t) + i(t))\times A$) around the mean total bacterial load. The mean is obtained by numerically solving the differential equations (1) and (2).

**Table 1:** Elementary reactions of logistic growth process.

Reaction	Type	Order	Rate constant	Rate constant units
$I\overset{d_1}{\longrightarrow }\phi$	Death	1	$d_1$	day$^{-1}$
$I\overset{b_1}{\longrightarrow }I + I$	Birth	1	$b_1$	day$^{-1}$
$I + I\overset{d_2}{\longrightarrow }I$	Death	2	$d_2$	bacteria$^{-1}$ day$^{-1}$
$I + I\overset{b_2}{\longrightarrow }I + I + I$	Birth	2	$b_2$	bacteria$^{-1}$ day$^{-1}$

Setting $b_2 = d_1 = 0$ (no density-dependent division + no density-independent death) results in a small variance around the mean S1+S2 (see Fig. 1c). Setting either $b_2$ or $d_1$ to nonzero values increases this variance dramatically. This increased variance increases the probability of the total bacterial load reaching 0, in which case the person will not develop disease. This result indicates that $b_2$ and $d_1$ serve as knobs that control variance in bacterial populations, to influence pathogenesis and dose-response outcomes. Increasing $b_2$ or $d_1$ increases the odds of the bacterial population going to 0 and causing no disease symptoms.

We took this idea further to estimate the response probabilities using the concept of individual effective dose or IED^26^, which we first illustrate conceptually. Individuals with a total bacterial load above IED are assumed to develop symptoms^16,27^. The total bacterial load is given by $(h(t) + i(t))\times A$ and IED is represented by $i_{\text {thresh}}$ (units CFU) (see Fig.1d). Individuals in whom bacteria die out completely are assumed to be unaffected. Individuals who fit neither category, in whom the bacterial load takes an intermediate value, are assumed to be the asymptomatic carrier population. Here, *h*(*t*) and *i*(*t*) (units CFU/cm$^{2}$) are multiplied by *A* to match the units of $i_{\text {thresh}}{}$.

Here we have assumed bacteria in the un-adjusted S1 state contribute to the response probabilities. This assumption is valid if the contribution of *h*(*t*) to the sum $h(t)+i(t)$ is small. This is the case if 1) bacteria in S2 are growing while bacteria (see Eq. (2)) in S1 are dwindling (see Eq. (1)) in numbers, with passing time and 2) bacteria in S1 die faster than bacteria in S2 grow ($r_1 > r_2$).

This concept is implemented in practice by repeatedly performing stochastic simulations. The probability of showing symptoms or response ($\hat{P}_{\text {res}}$) is found by calculating the fraction of simulations where total bacterial load exceeds $i_{\text {thresh}}$ as represented by Eq. (4)

4 $$\begin{aligned} \hat{P}_{\text {res}}{} = \frac{\text {no. of simulations with } (h(t) + i(t)) A\ge i_{\text {thresh}}{}}{n} \end{aligned}$$

where *n* is the number of stochastic simulation repetitions performed.

### Parameterizing 2C model for SA

We parameterized the model with data from the Singh et al. study^15^. Singh et al. performed a clinical trial wherein the participants’ hands were cleaned with alcohol and inoculated with a known dose of SA. The area was covered immediately with a patch of polyethylene film to uniformly distribute the inoculum underneath it. Bacterial densities in the covered area were measured over 6 days (growth data). This was used to identify the parameters $r_1$, $r_2$, $r_3$ and $i_{\text {max}}$ by minimizing the sum of squared errors in the $\log _{10}$ bacterial loads as given below.

5 $$\begin{aligned} f_{\text {SSE}}= \text {min.} \sum _{c} \sum _{j} \bigg (\log _{10}\big (h_c(t_j) + i_c(t_j)\big ) - \log _{10}\big (y_c(t_j)\big )\bigg )^2 \end{aligned}$$

Here $f_{\text {SSE}}$ is the minimized objective function value and *y* is the observed SA density. The subscript *c* sums over the 3 different initial inoculating densities used in Singh et al.’s experiment while the subscript *j* sums over the observation time points. Since initially none of the bacteria have adjusted to the host, we set *i*(0) to 0 and *h*(0) to the initial inoculating density i.e., $h_c(0) = y_c(0)$. Using this objective function assumes that both un-adjusted and adjusted cells are picked up while taking measurements.

Singh et al. also counted the number of people who developed lesions by day 6 for a given dose (dose-response data, see Supplementary Table S1). These data were used to identify the rate parameters ($b_2$ and $d_1$) and the IED ($i_{\text {thresh}}$). For this, the response probability was estimated using Eq. (4). This predicted response probability ($\hat{P}_{\text {res}}$) was used to minimize the deviance^13^ given below.

6 $$\begin{aligned} f_{\text {dev}}= \text {min.} \sum _j -2\bigg ( \hat{n}_{\text {res}}{}_{,j} \log { \bigg ( \frac{P_{\text {res}}{}_{,j}}{\hat{P}_{\text {res}}{}_{,j}} \bigg )} + (\hat{n}_{\text {tot}}{}_{,j} - \hat{n}_{\text {res}}{}_{,j}) \log { \bigg ( \frac{ 1-P_{\text {res}}{}_{,j} }{ 1-\hat{P}_{\text {res}}{}_{,j} } \bigg )} \bigg ) \end{aligned}$$

Here $f_{\text {dev}}$ is the minimized deviance. The subscript index *j* distinguishes the different SA doses that were administered. $\hat{n}_{\text {res}}$ is the total number of people showing response for a given dose of SA. $P_{\text {res}}$ and $\hat{P}_{\text {res}}$ are the observed and predicted response probabilities. $\hat{n}_{\text {tot}}$ is the total number of people given a particular dose of SA. The dose-response data was verified to exhibit a trend according to the one-tailed Cochran-Armitage test^13^ with $Z_\text {CA}=6.29$ (P$=1.55\times 10^{-10}$, n=6, significance level $=0.05$). Minimization of Eq. (6) was carried out by an optimization approach outlined in the Methods.

Some points of note are: (1) The probabilities of unaffected and carrier outcomes are computed by their corresponding fractions of simulations i.e., simulations with zero load and simulations with intermediate load ($0<(h(t) + i(t))A< i_{\text {thresh}}{}$). (2) The *b* and *d* values discussed in this paper correspond to stochastic rate constants ($c_\mu$ as discussed in Gillespie’s paper^25^) and not the standard (deterministic) rate constants. Stochastic rate constants are used for simulating bacterial numbers (CFU) with CTMC, whereas standard rate constants are used for simulating concentrations or densities (CFU/cm$^{2}$) with differential equations. (3) $i_{\text {max}}$, the carrying capacity, is interpreted as the mean of the SA densities observed in a population of individuals in whom SA is not wiped out. It differs from the IED ($i_{\text {thresh}}$), which is interpreted as the SA load above which an individual from the population will develop skin lesions.

In summary, we used the data from SA growth on the skin to identify the parameters of the 2C model’s differential equations (Eq. (1), (2) which are $r_1$, $r_2$, $r_3$ and $i_{\text {max}}$). And we use these parameters, along with the data on dose-response for skin lesions, to identify the parameters of the 2C models’ logistic growth process (which are $b_2$, $d_1$ and $i_{\text {thresh}}$).

### Assessing deterministic model fit

We first present the fit of the 2C model to SA growth data and the determination of the parameters $r_1$, $r_2$, $r_3$ and $i_{\text {max}}$. We used a Bayesian algorithm for fitting these parameters, which provided samples from the posterior distributions of the parameters. These samples differed in parameter combinations, and how well they fit the growth data (given by $f_{\text {SSE}}$, see Eq. (5)). A subset of the outcomes was used to identify the parameters of the logistic growth process. Specifically, we used the top 100 samples ranked by $f_{\text {SSE}}$, and also present the quality of these for completeness.

**Figure 2:**
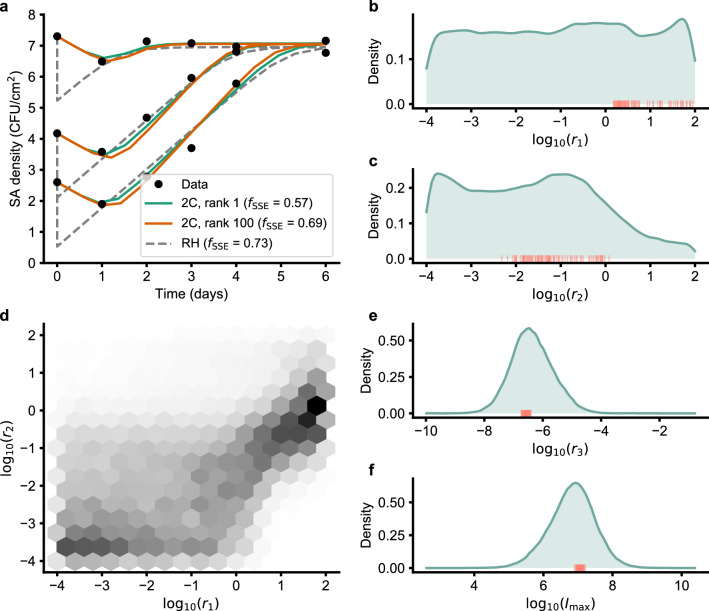
2C deterministic model fit and parameters. (**a**) 2C model fit to growth data. (**b**, **c**, **e**, **f**) Posterior distribution of parameters with top 100 solutions ranked by objective value Eq. (5). (**d**) Joint distribution of the posteriors of $r_1$ and $r_2$ parameters.

As expected from its design, the 2C model recapitulates the broad trends in the growth data of Singh et al. as seen in Fig. 2a. These include the initial dip in SA density, followed by an increase and stabilization at a high SA density that is independent of the initial inoculation. Both rank 1 and rank 100 solutions by $f_{\text {SSE}}$ appear to fit the data well. This fit is better (lower value of $f_{\text {SSE}}$) than that exhibited by the earlier RH model^14^, which also exhibits a sharper dip.

Among the fitted parameters, $r_3$ and $i_{\text {max}}$ were identifiable while $r_1$ and $r_2$ were not tightly constrained by the data (Fig. 2b–f). The top 100 of these parameters are more tightly constrained, with $\log _{10}r_1$ largely lying in [0, 2] and $\log _{10}r_2$ largely lying in [-2, 0]. The joint posterior of $\log _{10}r_1$ and $\log _{10}r_2$ (Fig. 2d) shows a somewhat linear relationship between these parameters, with a majority of $r_1 > r_2$.

### Assessing stochastic model fit

**Figure 3:**
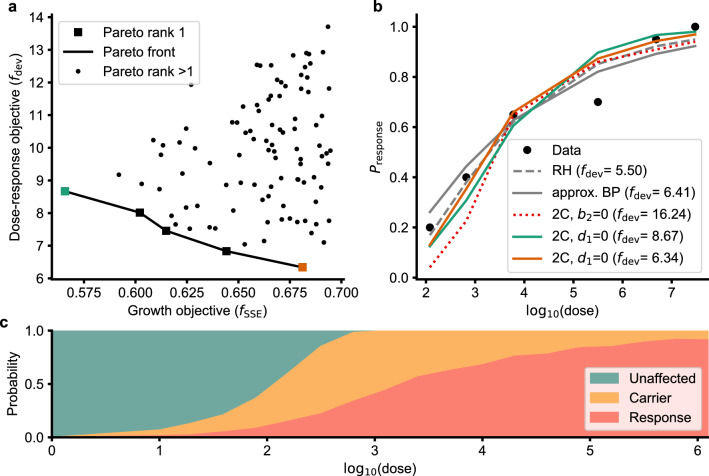
2C stochastic model fit and parameters. (**a**) Comparison of fits to the growth data ($f_{\text {SSE}}$) and dose-response data ($f_{\text {dev}}$). (**b**) Dose-response probabilities for the 2C model cases, along with RH and aBP models. Two colored 2C models with $d_1=0$ are the solutions at the extremes of the Pareto front in A. (**c**) Outcome probabilities as a function of dose for the 2C model with $d_1=0$ and $f_{\text {dev}}{} = 6.34$.

The stochastic component of the 2C model fits the dose-response data, with some samples performing better (or having lower $f_{\text {dev}}$) than others (Fig. 3a). This trade-off helps distinguish the suboptimal solutions (those solutions which have a higher $f_{\text {dev}}$ and a higher $f_{\text {SSE}}$ than some other solution) from the optimal ones. We refer to these optimal solutions as Pareto rank 1 solutions (squares in Fig 3a), which form a boundary referred to as the Pareto front.

The Pareto rank 1 solutions (Supplementary Table S2) presented above were found by setting $d_1=0$ & $b_2 >0$ in the 2C stochastic model. We compare two of these Pareto rank 1 solutions with other baseline models including: (1) the approximate beta-Poisson (aBP) model^13^, (2) the quasi-mechanistic DRM of Rose and Haas (RH model)^14^ and (3) the 2C model with $b_2=0, d_1>0$ with results presented in Table 2 and Fig 3b. The 2C model with $d_1=0$ fits the data as well as the aBP and RH models at the 0.05 significance level. Setting $b_2=0$ fails to fit the data at the 0.05 significance level, and this hypothesis is rejected. Since $b_2$ cannot be zero, we fail to reject the hypothesis that cooperation between individual SA cells is necessary for pathogenesis of SA . The unaffected, carrier and response probabilities for the 2C model with $d_1 = 0$ are presented in Fig. 3c. As expected with increasing dose, the unaffected probability decreases and the response (lesion) probability increases. The carrier probability increases to a maximum around a dose of $10^3$ CFU before dwindling.

**Table 2:** Summary of model fits.

Model	$f_{\text {dev}}$	$\chi ^2_{\text {degrees}, 0.05}$	P	Conclusion
2C($d_1=0$)	6.34	$\chi ^2_{4, 0.05} = 9.49$	0.18	Fail to reject
2C($b_2=0$)	16.24	$\chi ^2_{4, 0.05} = 9.49$	2.71$\times 10^{-3}$	Reject
approx. Beta-Poisson	6.40	$\chi ^2_{4, 0.05} = 9.49$	0.17	Fail to reject
RH model	5.50	$\chi ^2_{5, 0.05} = 11.07$	0.36	Fail to reject

### Parameters in the absence of alcohol pre-treatment

As mentioned earlier, Singh et al.^15^ treated their subjects with alcohol before administering SA, effectively reducing the resident microflora load on the skin. In comparison, Rose and Haas^14^ reported an experiment similar to Singh et al.^15^ but the subjects were inoculated with SA without alcohol pre-treatment (skin was only cleaned by soap 24 hours before the experiment). We postulate the skin microflora in the Rose and Haas experiment exerts a competitive pressure that acts as a constant first order death rate on SA.

To determine the fit of 2C model to this more realistic scenario, we explore the model fit under the following two hypotheses. The first hypothesis (which we call $r_1^*$) assumes that only the un-adjusted SA are affected by the resident microflora (see Supplementary Methods, Supplementary Fig. S1a, S1c). The second hypothesis (which we call $r_{\text {mf}}$) assumes that both un-adjusted and adjusted SA are affected by resident microflora to an equal extent (see Supplementary Methods, Supplementary Fig. S1b, S1d). We note that other hypotheses may also fit the data from Rose and Haas^14^. However, we restrict ourselves to the hypotheses that can be modeled with one additional parameter. This is because there are only 4 data points including the initial condition. Using 2 parameters to fit the 3 remaining data points will result in over-fitting. For example, the corrected Akaike Information Criterion (AICc, which corrects for small sample sizes)^28^ for such a case (3 data points, 2 parameters) will be infinity. The limited data also hindered testing of absolute goodness of fit. Other hypotheses that can be captured by a single parameter change (e.g., resident microbiota affecting $r_2$) did not yield good fits (data not presented).

**Figure 4:**
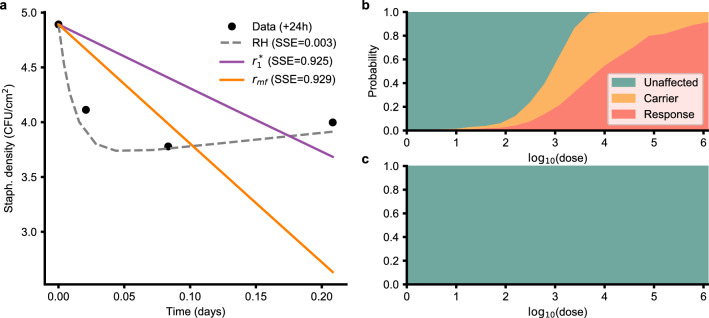
2C model in the absence of alcohol pre-treatment. Two different hypotheses ($r_1^*$ and $r_{\text {mf}}$, described in text) were investigated. (**a**) Fit of the two hypotheses to data from Rose and Haas^14^. (**b**) Outcome probabilities for $r_1^*$ hypothesis. (**c**) $r_{\text {mf}}$ hypothesis respectively.

The fit of these two hypotheses, along with the approach of Rose and Haas^14^ are compared in Fig. 4a. The RH model fits the data better because of the additional parameter. The $r_1^*$ hypothesis shows a sharper decline than the $r_{\text {mf}}$ hypothesis, but the available data does not strongly support either hypothesis as indicated by their similar SSE values.

The difference between the hypotheses is striking when looking at their outcome probabilities with increasing dose. The $r_1^*$ hypothesis predicts a significant $P_{\text {res}}$ at higher doses (Fig. 4b) whereas the $r_{\text {mf}}$ hypothesis predicts almost zero $P_{\text {res}}$ and carrier probability (Fig. 4c). This is because the adjusted SA also dies out from the inhibiting effect of the resident microflora.

## Discussion

A 2C model for SA dynamics on the human skin was developed and fitted to data on SA pathogenesis. By assuming that SA transitions from an un-adjusted state to an adjusted state, the model is grounded in first principles. The stochastic aspect of SA dose-response emerges naturally from a stochastic simulation of the growth kinetics. In addition, the model predicts carrier outcomes without additional data.

The 2C model fits the data on SA pathogenesis well, which is similar to the fit of the classical aBP DRM and the RH model. Yet, these models differ in their assumptions and the underlying biological mechanism they represent. For instance, the aBP assumes within-host variability of bacterial infectiousness and that the net bacterial load on an individual decreases with time. This decrease in bacterial numbers happens until the infection begins (initiated by one pathogen - the single hit hypothesis), and the model does not account for the SA regrowth observed in the kinetic data^15^ thereafter. The quasi-mechanistic RH model accounts for SA regrowth, and applies the exponential model to a revised dose which includes regrowth. This revised dose accounts for duration of SA presence on the skin explicitly. The 2C model accounts for the duration implicitly, since any SA that stays longer on the skin is likely to seed the explosive growth around the 3 day mark.

A second way in which the biological underpinnings of the 2C model differ from the aBP and RH models has to do with the cooperation hypothesis. The aBP stems from the single hit hypothesis and assumes complete independence between the bacteria in causing infection. The RH model does not assume complete independence between the bacteria - the logistic term assumes that the SA interact with each other to limit their growth rate, resulting in a negative feedback loop. An example of this is the competition for a limited resource such as physical space, nutrients, etc. in the stationary phase. In addition to this negative feedback loop, the 2C model with $b_2 >0$ posits the existence of a positive feedback loop. That is, there is some mechanism by which SA cooperate to produce a response, and the magnitude of this response is greater than that expected from the sum of the contributions of each bacterium. We note here that the cooperation discussed herein differs from the classical interpretation of cooperation in the context of the multi-hit hypothesis^13^. The multi-hit hypothesis posits that the barrier to infection is slowly broken down by the accumulated stress from multiple infectious pathogen units. There is no notion of direct cooperation between different infectious units as in the 2C model.

One possible explanation for the cooperative behavior above is the existence of one or more molecular pathways with signaling molecules that enhance growth. In addition, the *agr* quorum sensing system^29–31^ and the LexA/RecA SOS response system^32,33^ activate virulence genes to enhance pathogenesis and likely play a role. Hence it is possible to interpret the S2 compartment as a mathematical abstraction that accounts for factors that increase pathogenicity e.g. toxin production. Of course, other hypotheses such as within-host variability (as modeled by the aBP dose-response model) are also sufficient to explain the dose-response data. For future validation of the cooperation hypothesis, we suggest a dose-challenge experiment involving two treatments in a suitable host system. In the first treatment, a known dose of SA (say $N_1$) is applied to a known area of $A_1 \text {cm}^2$. In the second treatment, the same dose of SA is applied to a larger area $A_2$ such that $A_2 > A_1$. If there is cooperation between the bacterial units (as predicted by the 2C model), one would expect more responses (such as development of lesions in the host) in the first treatment. A few challenges arise such as ensuring uniform dispersal of SA in the inoculating dose. Additionally, there should be sufficient difference between $A_1$ and $A_2$ such that the posited signal can diffuse in treatment 1 but is diffusion-limited in treatment 2. The effects of between-host variability and acquired immunity can be reduced by performing both treatments simultaneously in each host.

We believe that the 2C model proposed here is a natural extension of our previous work—the Simple Death dose-response model (SD)^17^. Both models attempt to derive dose-response in a host as a consequence of stochastic kinetics of the pathogen in the host. This stochastic kinetics is modeled with CTMC, and response probability is evaluated based on the outcomes from a large number of stochastic simulations. A kinetics-based approach also allows us to apply the same DRM to antibiotic-resistant counterparts of pathogens, with the underlying assumptions being clear and intuitive. For example, we investigated the outcome probabilities for patients in a hospital where the bedrails are contaminated with MRSA (see Supplement). The results largely depend on the choice of hypothesis ($r_1^*$ or $r_{\text {mf}}$), assuming that MRSA kinetics on the skin mirrors that of its methicillin-sensitive counterpart. If there is evidence of greater (or lesser) virulence of a MRSA strain, perturbations to the kinetic parameters, such as a higher $r_3$ or lower $i_{\text {thresh}}$, may be investigated. So far, there has not been an effective model to predict the risk of MRSA. The 2C model framework provides a unique opportunity for MRSA risk computation.

Some key differences between the 2C and SD DRM include the underlying kinetic processes considered, and the final form of the model. The SD DRM only accounts for bacterial die-out and predicts response if the bacteria survive past a threshold time point. The 2C model does not define such a threshold time point, and predicts response if pathogen numbers explode and cross a defined value (the IED). This also allows the 2C model to elegantly recover the carrier probability by evaluating the simulations.

This power of the 2C model comes at the price of challenges in implementing and parameterizing the 2C model. The SD model has an analytical expression which is trivial to evaluate and fit given traditional dose-response data. In comparison, the 2C model needs a large number of stochastic simulations to evaluate its parameters. This task is certainly computationally intensive, but modern clusters and software allow large-scale parallelization to navigate this challenge. Perhaps a bigger impediment to the 2C model is its requirement for kinetic data (in addition to dose-response data) to evaluate its parameters, which are not easily available for other pathogens. Parameterizing the 2C model for SA in the absence of alcohol pre-treatment was hindered by limited data as well. Experiments generating relevant kinetic data, and analytical expressions/approximations for the response and carrier probabilities of the 2C model are topics for further research.

## Supplementary information

Supplementary material 1 (pdf 1773 KB)

## Data Availability

Codes reproducing the results in this publication are available on GitHub at https://github.com/JiangLabUCI/TwoCompartment.
