# Supplementary material for: A dose response model for Staphylococcus aureus
Source: Sci Rep. 2021 Jun 15;11:12542. doi: 10.1038/s41598-021-91822-y (PMC8206448; doi:10.1038/s41598-021-91822-y)
Supplement: Supplementary file 1 — Supplementary material 1 (pdf 1773 KB) [file 41598_2021_91822_MOESM1_ESM.pdf]

A dose response model for *Staphylococcus aureus*

Srikiran Chandrasekaran[1,*] Sunny C. Jiang[1]

[1]University of California Irvine, Civil and Environmental Engineering, Irvine, 92697, United States

[*]Corresponding author (srikiranc@gmail.com)

Supplementary Methods

Equations of hypotheses for cases without alcohol pretreatment

The r_1^* hypothesis is given by the equations

$$\frac{dh(t)}{dt} = -r_1^* h(t) - r_2 h(t) \tag{1}$$

$$\frac{di(t)}{dt} = r_2 h(t) + r_3 i(t)(i_{\max} - i(t)) \tag{2}$$

Here r_1^* reflects the increased r_1, accounting for death by resident microflora. The r_{mf} hypothesis is given by the equations

$$\frac{dh(t)}{dt} = -r_1 h(t) - r_2 h(t) - r_{\mathrm{mf}} h(t)$$

$$\frac{di(t)}{dt} = r_2 h(t) + r_3 i(t)(i_{\max} - i(t)) - r_{\mathrm{mf}} i(t)$$

Case study

As an example, we look at the bedside rails in a hospital as an environmental reservoir of MRSA and the subsequent risk to a patient using that bed. The patient's exposure to MRSA is calculated as the product of the MRSA density/cm^2 on bed-rails [1], area of hands [2] and the transfer efficiency from bed-rails to hands [3] (see assumptions and implementation details below). Exposure is assumed to randomly occur at a hand-bedside rail contact frequency of 0.28 contacts/hour [4]. The environmental reservoir is assumed to not be affected by contact events, which is possible if the patient touches different parts of the bedside rails. In this way, the MRSA load in a patient staying in a hospital was modeled with stochastics. This was repeated 1000 times to understand the probabilities of different outcomes.

Sample trajectories of the MRSA load on the patient over time is presented in Fig. S1A (r_1^* hypothesis) and S1B (r_{mf} hypothesis). The sudden increases in MRSA loads are indicative of the random contact events. This is usually followed by a period of decay. In some cases, when the populations cross 10^4 CFU, the contact events are not discernible from the general randomness in the simulation. Some trajectories under the r_1^* hypothesis undergo explosion while none of those under r_{mf} do. The sharp increase at the ends of the explosive trajectories (Fig. S1A, red lines) highlight the numerical difficulties in simulating stochastic systems with large number of entities.

The distribution of outcome probabilities is given in Fig. S1C (r_1^*) and S1D (r_{mf}). Initially, both hypotheses predict similar outcomes. At the end of day 1, they start to diverge with r_1^* predicting an increasing likelihood of carrier outcomes and even predicting response outcomes by day 6. On the other hand, r_{mf} predicts a more or less constant probability of carrier status and no response outcomes through day 6 (Fig. S1D). The corresponding trajectories (Fig. S1B) explain this behavior, as MRSA loads from exposure events are not large enough to overcome the decay and establish response. This is consistent with the trend seen earlier (Fig. 4) of r_{mf} predicting less adverse outcomes than r_1^*.

Overall, we see that the 2C model enables simulation of repeated exposure without needing the assumption of independence between exposures. In fact, accounting for the net exposure uncovers the

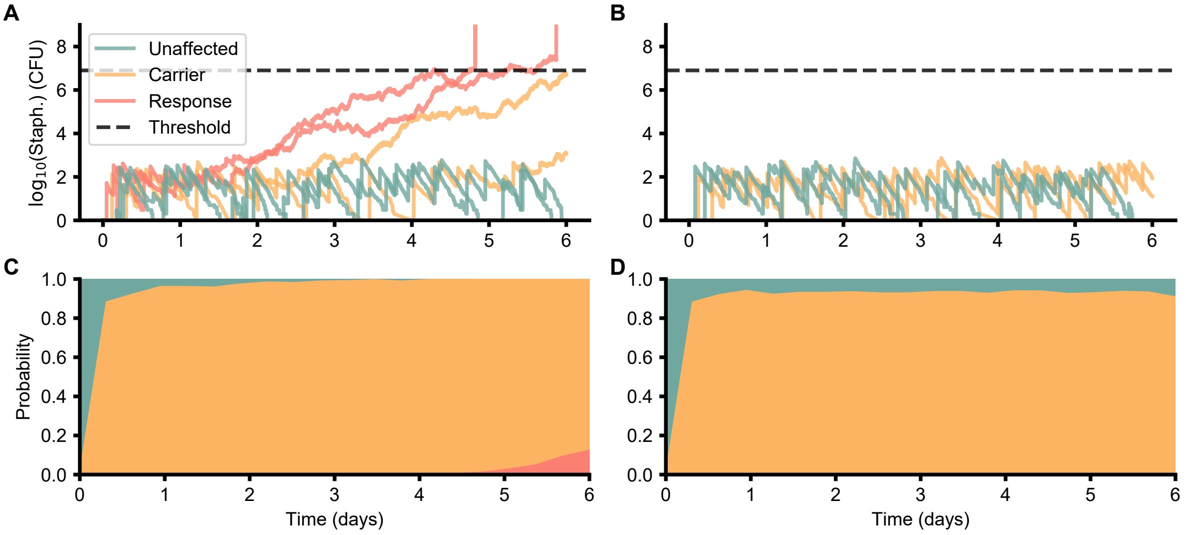

Figure S1: Case study (described in text) outcomes for the r_1^* (A and C) and r_{mf} (B and D) hypotheses. A and B show MRSA population trajectories for the r_1^* and r_{mf} hypothesis, respectively. $\log_{10}$ (SA population + 1) is plotted to avoid (negative) infinities. C and D show the outcome probabilities for the r_1^* and r_{mf} hypothesis, respectively. Color labels are as indicated in A. The horizontal dashed line in A and B represents the IED used to distinguish between Carrier and Response outcomes.

potential increase in response probability over extended periods (Fig. S1A). The outcomes predicted by the two hypotheses (r_1^* and r_{mf}) vary, and a possible avenue of future research is collecting more data to test these hypotheses and their alternatives. Additionally, insights into the kinetics of MRSA in relation to MSSA will help relax the assumption of same kinetic parameters for both MRSA and MSSA.

Assumptions and implementation details

Patients were assumed to occupy the same bed for the duration of 6 days. Exposure to MRSA was modeled as a Poisson process and assumed to occur at random with a contact frequency of 0.28 contacts/hour [4]. Hence, the time between two exposure events was sampled from an exponential distribution with rate parameter = 0.28 contacts/hour.

Each exposure event resulted in an increase in the number of SA in S1. This inoculation load or increase was computed as the product of 1) MRSA density/cm^2 on bed-rails ([1], sampled from a normal distribution with mean = 159.5 CFU/100 cm^2, std. dev. = 396.4 CFU/100 cm^2, truncated at (0, 1620)), 2) area of hands ([2], women's hand size = 132.42 cm^2), 3) transfer efficiency from bedside rails to hands ([3], sampled from a uniform distribution with bounds [0.22, 0.38]). At each exposure time, the simulation was halted and the number of SA in S1 was increased by the inoculation load.

Dose response data

Table S1: Dose response data from [5]

Inoculation density (CFU/cm^2)	$\hat{n}_{\mathrm{res}}$	$\hat{n}_{\mathrm{tot}}$	$\hat{P}_{\mathrm{res}}$
40	4	20	0.20
220	8	20	0.40
2000	13	20	0.65
105000	14	20	0.70
1600000	19	20	0.95
10000000	20	20	1.00

Rank 1 solutions

Table S2: Rank 1 solutions in incresing order of growth-objective

r_1 (/day)	r_2 (/day)	r_3 (cm^2/(CFU day))	$i_{\max}$ (CFU/cm^2)	b_2 (/day)	i_{thresh} (1/(CFU day))	f_{SSE}	f_{dev}
1.94	1.47e-02	2.71e-07	1.18e+07	1.70	7.34e+06	0.57	8.67
1.90	1.55e-02	3.56e-07	8.93e+06	2.00	4.81e+06	0.60	8.01
2.10	2.68e-02	2.92e-07	1.05e+07	0.70	2.04e+07	0.61	7.46
2.19	1.95e-02	3.53e-07	9.09e+06	1.80	6.88e+06	0.64	6.83
1.69	1.02e-02	3.91e-07	8.70e+06	1.70	7.97e+06	0.68	6.34

References

[1] Kurashige, E. J. O., Oie, S. & Furukawa, H. Contamination of environmental surfaces by methicillin-resistant Staphylococcus aureus (MRSA) in rooms of inpatients with MRSA-positive body sites. *Brazilian Journal of Microbiology* **47**, 703–705 (2016). URL `http://dx.doi.org/10.1016/j.bjm.2016.04.002`.

[2] Agarwal, P. & Sahu, S. Determination of hand and palm area as a ratio of body surface area in Indian population. *Indian journal of plastic surgery : official publication of the Association of Plastic Surgeons of India* **43**, 49–53 (2010). URL `http://www.ncbi.nlm.nih.gov/pubmed/20924450http://www.pubmedcentral.nih.gov/articlerender.fcgi?artid=PMC2938623`.

[3] Ali, S., Moore, G. & Wilson, A. P. R. Effect of surface coating and finish upon the cleanability of bed rails and the spread of Staphylococcus aureus. *Journal of Hospital Infection* **80**, 192–198 (2012). URL `http://dx.doi.org/10.1016/j.jhin.2011.12.005`.

[4] Cheng, V. C. *et al.* Hand-touch contact assessment of high-touch and mutual-touch surfaces among healthcare workers, patients, and visitors. *Journal of Hospital Infection* **90**, 220–225 (2015).

[5] Singh, G., Marples, R. R. & Kligman, A. M. Experimental Staphylococcus Aureus Infections in Humans. *Journal of Investigative Dermatology* **57**, 149–162 (1971). URL `http://www.ncbi.nlm.nih.gov/pubmed/4937674http://linkinghub.elsevier.com/retrieve/pii/S0022202X15480418`.
